# Analysis of the Behaviour of Immunoglobulin G Antibodies in Children and Adults Convalescing From Severe Acute Respiratory Syndrome-Coronavirus-2 Infection

**DOI:** 10.3389/fped.2021.671831

**Published:** 2021-08-17

**Authors:** Horacio Márquez-González, Briceida López-Martínez, Israel Parra-Ortega, Daniela de la Rosa-Zamboni, Marcela Salazar-García, Victor Olivar-López, Miguel Klünder-Klünder

**Affiliations:** ^1^Clinical Research, Hospital Infantil de México Federico Gómez, Mexico City, Mexico; ^2^Auxiliary Diagnostic Resources, Hospital Infantil de México Federico Gómez, Mexico City, Mexico; ^3^Clinical Laboratory, Hospital Infantil de México Federico Gómez, Mexico City, Mexico; ^4^Clinical Epidemiology, Hospital Infantil de México Federico Gómez, Mexico City, Mexico; ^5^Biomedical Research, Hospital Infantil de México Federico Gómez, Mexico City, Mexico; ^6^Pediatric Emergency Service, Hospital Infantil de México Federico Gómez, Mexico City, Mexico; ^7^Management of Clinical Research, Hospital Infantil de México Federico Gómez, Mexico City, Mexico

**Keywords:** SARS-CoV-2, COVID-19, antibobies, paediatric population, seroprevalance, Mexico

## Abstract

The pandemic caused by SARS CoV-2 (COVID-19) has affected millions of people since 2020. There are clinical differences and in mortality between the adult and paediatric population. Recently, the immune response through the development of antibodies has gained relevance due to the risk of reinfection and vaccines' development.

**Objective:** Was to compare the association of clinical history and the clinical presentation of the disease with the development of IgG antibodies against SARS-CoV-2 in paediatric and adult patients with a history of positive reverse transcriptase-polymerase chain reaction (RT-PCR) results.

**Methods:** Cross-sectional observational study carried out in a Paediatric Hospital in Mexico City included patients under 18 years of age and health personnel with positive RT-PCR for COVID-19 comparing antibody expression. The development of specific IgG antibodies was measured, the presence of comorbidities, duration, and severity of symptoms was determined.

**Results:** Sixty-one subjects (20 < 18 years and 41 > 18 years) were analysed. The median sample collection was 3 weeks. There were no differences in the expression of specific antibodies; no differences were shown according to the symptoms' severity. A positive correlation (*r* = 0.77) was demonstrated between the duration of symptoms and antibody levels.

**Conclusions:** In conclusion, there is a clear association between the duration of the symptoms associated with SARS-CoV-2 infection and the IgG units generated in paediatric and adult patients convalescing from COVID-19.

## Introduction

Coronavirus disease-2019 (COVID-19), the pandemic caused by the new strain of coronavirus called severe acute respiratory syndrome coronavirus-2 (SARS-CoV-2) has affected more than 200 countries, and until February 2021, it has affected >11 million people causing >2,000,000 deaths (1).

The disease's behaviour is mainly characterised by respiratory symptoms, which can be complicated with severe respiratory insufficiency. It has an incubation period of 3–14 days from exposure, and a duration of 1–2 weeks (2).

The generation of specific immunoglobulin G (IgG) antibodies against SARS-CoV-2 is triggered by the host's immune response and is directly related with the decrease in symptoms as well as the protection against future exposures (3). IgG antibodies are particularly relevant in this disease to explain the effectiveness of vaccines. Seroprevalence studies have demonstrated that the expression of antibodies is directly related with the prevalence of the disease (4).

The effect of age on the susceptibility to infection, clinical manifestations and development of complications is already known. In this regard, most publications predominantly study the paediatric population without comorbidities, and thus, it is useful to analyse the response of the immune system of children with previous illnesses as well as that of the healthcare personnel caring for them (5). The objective of this study was to compare the association of clinical history and the clinical presentation of the disease with the development of IgG antibodies against SARS-CoV-2 in paediatric and adult patients with a history of positive reverse transcriptase-polymerase chain reaction (RT-PCR) results.

## Materials and Methods

In a third-level paediatric hospital in Mexico City (June to September 2020), converted to a COVID-19 paediatric centre, we conducted a comparative cross-sectional study in healthcare personnel and paediatric patients with a history of SARS-CoV-2 infection in the convalescent stage. The ethics committee approved the protocol.

The study included subjects with a history of symptoms or who have been in contact with a confirmed case of SARS Cov-2), of any age with a positive RT-PCR confirmatory test for SARS-CoV-2, a documented follow-up of the disease's evolution and measurement of IgG antibodies, who had confirmed their participation by signing an informed consent form.

Two comparison groups were made based on age: subjects < 18 years of age, who comprised the population of patients being treated in the hospital, and subjects > 18.1 years of age, recruited among infected healthcare personnel who were diagnosed and followed up by the Department of Occupational Medicine and Epidemiology.

Clinical data were obtained from all the study subjects and the convalescent stage was confirmed, defined as the absence of symptoms for more than 15 days in mild cases, and with negative RT-PCR test results for SARS-CoV-2.

Comorbidities (arterial hypertension, diabetes, obesity, and cancer), initial symptoms and vaccination history (obtained from the medical records of the patients <18 years of age and by direct questioning in those patients >18 years) were recorded. The history of the BCG vaccine was corroborated with the evidence of a scar on the arm and the record in the vaccination record.

The evolution regarding the need for hospitalisation, intensive care unit (ICU) and mechanical ventilation was recorded.

In all subjects, the diagnosis was performed using nasopharyngeal swabs in a Hanks Balanced Salt Solution. The extraction of ribonucleic acid (RNA) was carried out in an automated way with the QIAamp® Viral RNA Mini Accessory Set kit and the QIAcube Connect equipment. The viral genes ORF1ab, N, S, and the human RNase P gene (which allows for the assessment of the quality of the sample) were detected using the following commercial kits: Detection kit for 2019 Novel Coronavirus (2019-nCoV) RNA (PCR-Fluorescence Probing) Cat. DA-930 (Daan Gene Co., Ltd., Sun Yat-Sen University) and Applied Life Technologies Corporation, Thermofisher® Cat. A47532, in the thermocyclers QuantStudio 5 Real-Time PCR Systems, ThermoFisher® and CFX96 Real time system, C1000, Touch Thermal cycler, BIORAD®, following the manufacturers' recommendations and the current operational guidelines established by the Institute of Epidemiological Diagnosis and Reference (InDRE, by its Spanish acronym).

The detection of antibodies was carried out in peripheral blood using chemiluminescence microparticle immunoassay (CMIA). IgG class antibodies against the SARS-CoV-2 nucleocapsid protein were detected using an automated equipment ARCHITECT 11000SR, Abbott®, and values above 1.4 units were considered positive.

### Statistical Analysis

Qualitative variables were expressed in frequencies and percentages and quantitative variables in medians and interquartile ranges (Kolmogorov–Smirnov test demonstrated non-parametric behaviour of the variables). A comparison test was performed using Chi-squared test and Fisher's exact test. The Mann**–**Whitney *U*-test was used to measure the quantitative variables. The correlation between quantitative variables (duration of symptoms) and the expression of IgG was analysed using the Pearson test; a value of *R*^2^ > 0.7 being considered a “good correlation.” The statistical programme used was SPSS version 20 for MAC.

## Results

During the study period, 61 study subjects with a positive RT-PCR test result for SARS-CoV-2 were included: among these, 35 (57%) were women, and 41 (67%) were >18 years.

At the time of the study, IgG antibodies were expressed within positive ranges in 24 (39%) patients, showing no differences based on sex or the age group of the patients. The median time between those patients who expressed and did not express IgG antibodies was 23 days, and the statistically significant variables were the duration of symptoms and the persistence of fever for more than 3 weeks (Table 1).

**Table 1:** Differences between patients with and without IgG antibodies to SARS-CoV-2.

	**IgG SARS-CoV-2 antibodies**				***p***
	**No**		**Yes**		
	**37**		**24**		
	***n***	**%**	***n***	**%**	
Gender^*^					
Female	21	55	14	58	0.9
Male	16	45	10	42	
Age group^*^					
<18 years	10	27	10	42	0.9
>18 years	27	73	14	58	
Evolution^*^					
Ambulatory	23	62	9	38	0.2
Hospitalisation	14	38	15	63	
SARS-CoV-2 severe	9	24	6	25	0.2
Endotracheal intubation	3	8	14	42	**0.001**
Time^**^	**Median**	**p25–p75**	**Median**	**p25–p75**	
PCR positive (days)	29	(23–45)	18	(15–30)	**0.01**
Symptoms (days)	30	(25–37)	20	(15–42)	**0.03**
Symptoms (weeks)	1	(1–3)	3	(2–5)	**0.002**
Respiratory symptons	15	42	9	50	0.5
Fever > 3 weeks	9	25	11	57	**0.01**

* *X^2^ test*.

** *U Mann–Whitney. Bold values indicate statistical significance*.

**Figure 1:**
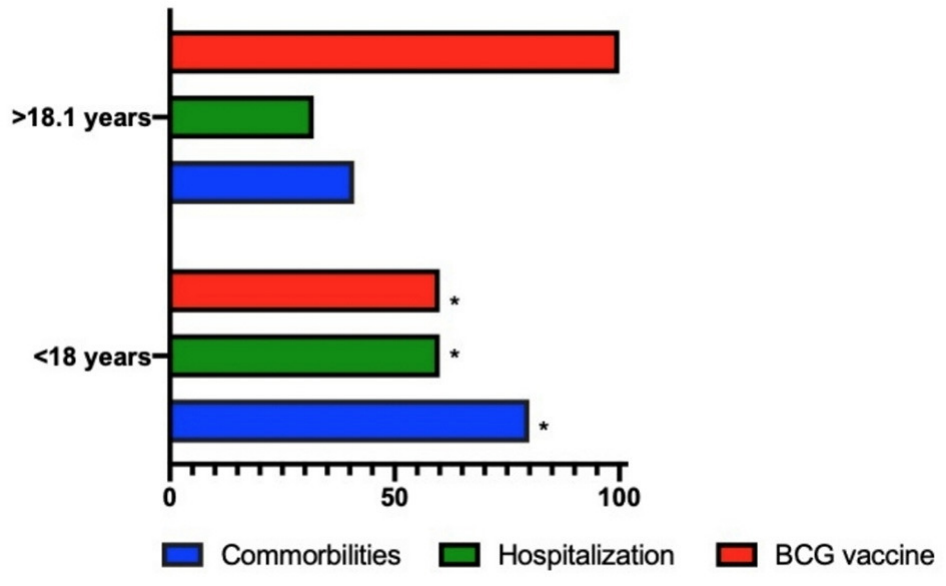
Differences between the paediatric population and health workers. *p value < 0.05.

In the comparison of the clinical characteristics by age group, no statistical difference was found in the variables of sex and duration of symptoms. Forty percent of paediatric patients jointly presented the virus with cancer. The variables with statistical differences were hospitalisation(Table 2), which was higher in the paediatric group (60 vs. 32%) and the Bacillus–Calmette–Guerin (BCG) vaccination history (100 vs. 60%) as well as the presence of any comorbidity (80 vs. 41%) which was higher in paediatric patients (Figure 1).

**Table 2:** Differences according to age group.

	** <18 years**		**>18 years**		p^*^
	**20**		**41**		
	***n***	**%**	***n***	**%**	
**Gender**					
Female	14	70	21	51	0.7
Male	6	30	20	49	
Time since symptoms (days)	24.5	10–43	30	23–42	0.6^**^
Time since RCP positive (days)	23	15–37	28	20–38	0.7^**^
Symptoms (weeks)	1	1–3	1	1–3	0.9^**^
IgG SARS-CoV-2 (units)	0.9	0.2–1.87	0.55	0.02–2.8	0.8
Hospitalisation	12	60	13	32	**0.02**
Endotracheal intubation	3	15	0	0	–
Influenza vaccine	3	15	9	22	0.7
**Commorbilities**	16	80%	17	41%	0.03
SAH	3	15	10	24	0.1
DM	1	5	3	7	0.9
Obesity	4	20	4	10	0.3
Cáncer	8	40	0	0	–

* *x^2^ test*,

** *U Mann–Whitney*.

*DM, Diabetes Mellitus; SAH, Systemic Arterial Hypertension. Bold values indicate statistical significance*.

**Figure 2:**
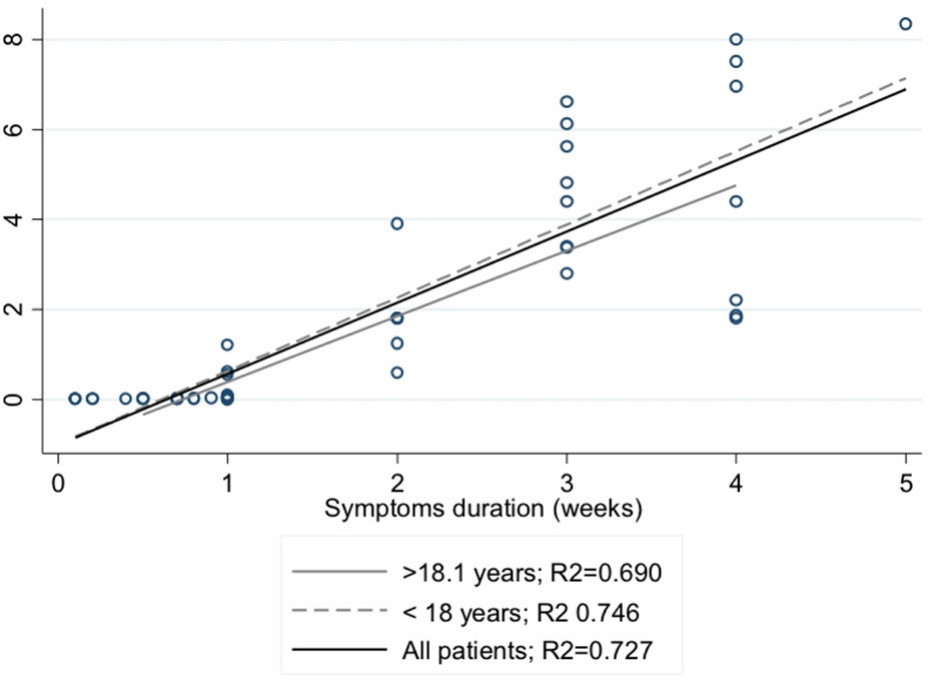
Correlation between duration of symptoms and levels of IgG for SARS-CoV-2.

**Figure 3:**
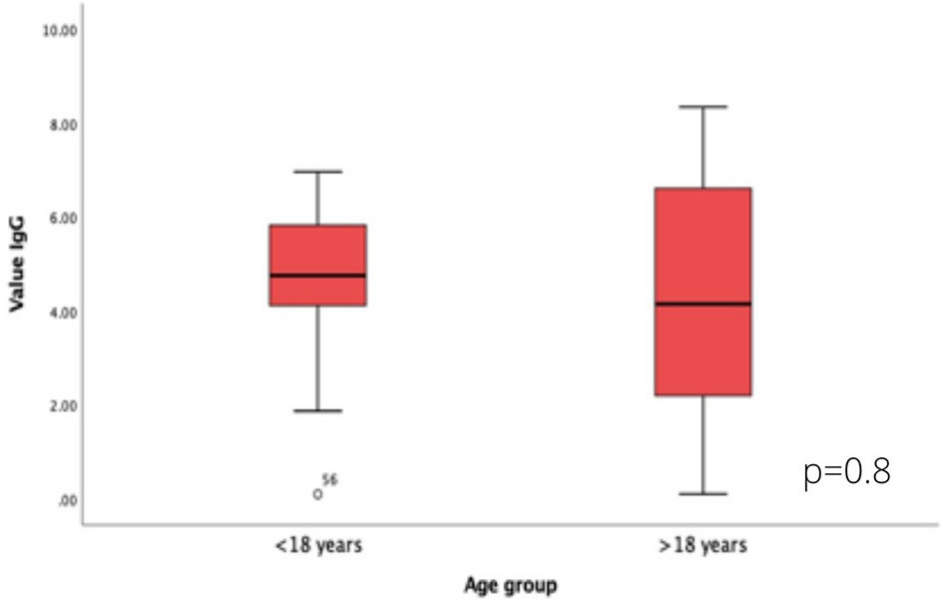
Differences in SARS-CoV-2 IgG values according to age group.

The simple linear regression analysis shows that the weeks of duration of the disease from the appearance of the first symptom in both groups accounts for *m* > 70% of the specific expression of IgG (*R*^2^ > 0.7) (Figure 2). No differences were shown in the quantitative value of the antibodies by age groups (Figure 3).

## Discussion

The present study demonstrates that there is an association between the duration of symptoms related to COVID-19 and the concentration of IgG antibodies against SARS-CoV-2 in both children and adults.

There is a relationship between the antibody expression and the clinical duration of the disease and its prevalence, as evidenced in the SEROCoV-POP study conducted in more than 1,000 volunteers, showing that the highest expression was of 10.6% at 3 weeks from the highest peak of positive subjects in that community. In this study, 41% of the subjects expressed specific IgG antibodies (6). However, all were confirmed cases and the median time at the time of determination was 23 (19–25) days after diagnosis by PCR.

Although the SEROCoV-POP study (6) found that the expression of antibodies was <0.8% in children under 10 years, it should be considered that this age group has been the least exposed group among all populations due to its lack of mobility (schools, kindergartens, and recreational centres). However, when this comparison is made within the hospital environment, the differences in expression are not related to age, but to the severity of the symptoms, as shown by Chen et al. (7) in a study analysing 12 adults and 20 subjects of paediatric age, in which no differences in the expression of immunoglobulin M (IgM) and IgG antibodies between both comparison groups were demonstrated. Similarly, in the present study, no differences in expression between children and adults (42 vs. 58%) were observed.

The group expressing antibodies presented a higher proportion of intubated patients (12 vs. 2.1%, *p* = 0.05) and severe manifestations due to COVID-19 (32 vs. 25%, *p* not significant). The clinical variables did not show differences between age groups. It should be noted that the paediatric population included in this study was recruited from a paediatric hospital centre that mainly cares for children with serious illnesses. This is reflected in the comparison by groups of age, in which 60% of patients were hospitalised, 40% of which were patients also suffering from cancer and undergoing treatment. The duration of symptoms was directly correlated with the expression of antibodies. This is consistent with a study by Gozalbo-Roviro et al. (8) in 32 patients reporting that the expression of antibodies was mainly associated with the temporality of the infection, regardless of whether the subjects required intensive care. Additionally, 64% of the subjects who did not express antibodies at the time of diagnosis were asymptomatic. Jiang et al. analysed a cohort of 214 infected subjects in order to evaluate seroconversion; the data show a median of 7 days (5–8) in the asymptomatic group (85% expressed specific IgG antibodies) compared to 24 (18–29) days (93% expressed IgG) in symptomatic patients (9). Symptoms have shown a poor relationship with the expression of antibodies, Cervia et al. (10) reported in 64 adult patients infected by SARS Cov-2 a positive correlation of specific IgG values with the severity of the disease (pneumonia, need for hospitalisation), days of symptoms and age. In our study, the median time between the result of the PCR and the antibody test was 23 days. In view of these findings, the proportion of subjects without IgG expression may be explained by the time interval in which the antibodies were obtained as well as by the presence of symptomatology.

The occurrence of the disease due to COVID-19 is different in the paediatric and adult populations, conditioning factors of which, include the different expression sites of angiotensin-converting enzyme 2 (ACE2) in children (11). Consequently, there is a greater proportion of gastrointestinal or diffuse symptoms, a situation that makes the early identification of the disease by caregivers as well as its timely registration particularly difficult.

In Mexico, the BCG vaccine is included in the national vaccination scheme; in this study, in patients older than 18 years, this history was proportionally higher (100 vs. 60%). During the first global wave of COVID-19 infections, a more severe and lethal behaviour was demonstrated in countries with a vaccination plan without including BCG compared to those with a positive history (12). One of the hypotheses regarding the effect of this vaccine suggests that there is an epigenetic reprogramming of monocytes to induce a pro-inflammatory response (production of cytokines iL-1β, TNF, and IL-6) in subsequent exposures to various viruses, achieving attenuation on the severity of the disease (13). In this work, this variable was not the primary objective; however, the need for hospitalisation in those over 18 years of age was statistically lower, but the history of the vaccine did not show a relationship with the expression of specific IgG antibodies for SARS Cov-2.

In addition, the greatest comorbidity in the paediatric group was haematologic cancer, in which the therapeutic target is the reduction of immature lymphocytes and which in healthy subjects are responsible for the generation of antibodies (14, 15).

It is important to consider that the study subjects were recruited within the setting of a paediatric hospital in which the children presented with mild symptoms to corroborate the diagnosis or were admitted for complications of the underlying condition and the coexistence of infection by COVID-19 (16, 17). The adult population were composed of the healthcare professionals who were in contact with them. In addition to representing a selection bias, it is possible that the viral load from the exposure of the adult group be lower due to the fact that only four children were classified as presenting severe disease, and the protective equipment enhanced that attenuation. In this regard, Chen et al. (7) conducted a cohort study in 52 patients with SARS-CoV-2 in which a positive correlation between the viral load and IgG antibody levels was found.

The results of this study allowed for the identification of a situation that could be different with regard to the behaviour of the pandemic within the context of exclusively paediatric hospitals. The entire study population were confirmed subjects of SARS-CoV-2 and the expression of antibodies was lower than that reported in other studies, which justifies the performance of longitudinal causality and prognosis studies that allow for the assessment of the interaction of confounding variables. The cross-sectional characteristics of this study make it potentially susceptible to temporality, memory, and misclassification biases. These results suggest continuing with the actions that prevent the spread of the virus even in infected people, who are not exempt from being infected again.

In conclusion, there is a clear association between the duration of the symptoms associated with SARS-CoV-2 infection and the IgG units generated in paediatric and adult patients convalescing from COVID-19. Additionally, those who developed these antibodies showed a more severe course of the disease, characterised by a longer duration of fever and the requirement of endotracheal intubation in a greater proportion of these patients.

## Data Availability Statement

The raw data supporting the conclusions of this article will be made available by the authors, without undue reservation.

## Ethics Statement

The studies involving human participants were reviewed and approved by Federico Gomez Children Hospital (HIM-031-2020). Written informed consent to participate in this study was provided by the participants' legal guardian/next of kin.

## Author Contributions

HM-G, MK-K, and MS-G organisation of data, analysis of results, writing, and contributions to the latest version of the document. BL-M, IP-O, VO-L, and DR-Z processing of samples, clinical examination of patients, and contributions to the newest version of the protocol. All authors contributed to the article and approved the submitted version.

## Conflict of Interest

The authors declare that the research was conducted in the absence of any commercial or financial relationships that could be construed as a potential conflict of interest.

## Publisher's Note

All claims expressed in this article are solely those of the authors and do not necessarily represent those of their affiliated organizations, or those of the publisher, the editors and the reviewers. Any product that may be evaluated in this article, or claim that may be made by its manufacturer, is not guaranteed or endorsed by the publisher.
